# A Comparative Analysis of Antimicrobial Resistance Patterns and Genes in *Staphylococcus aureus* From Humans and Animals in Veterinary Clinics Across Thailand

**DOI:** 10.1155/tbed/5541655

**Published:** 2025-07-21

**Authors:** Shutipen Buranasinsup, Anuwat Wiratsudakul, Sarin Suwanpakdee, Sineenard Jiemtaweeboon, Khuanwalai Maklon, Walasinee Sakcamduang, Boonrat Chantong

**Affiliations:** ^1^Department of Pre-Clinic and Applied Animal Science, Faculty of Veterinary Science, Mahidol University, Nakhon Pathom 73170, Thailand; ^2^Department of Clinical Sciences and Public Health, Faculty of Veterinary Science, Mahidol University, Nakhon Pathom 73170, Thailand; ^3^The Monitoring and Surveillance Center for Zoonotic Diseases in Wildlife and Exotic Animals, Faculty of Veterinary Science, Mahidol University, Nakhon Pathom 73170, Thailand

**Keywords:** antimicrobial resistance, occupational risk, pets, *Staphylococcus aureus*, Thailand, veterinarians

## Abstract

**Background:** Antimicrobial resistance (AMR) in *Staphylococcus aureus* (*S. aureus*) poses critical public health challenges by limiting treatment efficacy and elevating morbidity, mortality, and healthcare costs.

**Methods:** This study examined the prevalence and characteristics of AMR in *S. aureus* isolated from humans (veterinarians, veterinary assistants, and pet owners) and animals (dogs and cats) in veterinary clinics across five provinces in Thailand. A total of 882 samples were collected from which 188 *S. aureus* isolates were recovered and subjected to antimicrobial susceptibility testing and resistance gene detection.

**Results:** Substantial variations in AMR profiles were observed across host categories, with veterinarians and veterinary assistants exhibiting higher resistance rates than pet owners. The β-lactam resistance gene *bla*Z was prevalent in all groups, whereas *mec*A was predominantly detected in veterinarians and dogs, emphasizing the occupational risk and zoonotic transmission potential. The aminoglycoside resistance gene *aac*A*–aph*D was common in cats, and quinolone resistance genes *gyr*A and *grl*A were identified in veterinarians and dogs. Macrolide resistance genes *msr*A and *erm*A, lincosamide resistance gene *lin*A, and tetracycline resistance gene *tet*K were widely distributed across the groups. *Agr* typing of *S. aureus* isolates revealed diverse group distributions, with *agr* group I was predominant in human samples and associated with the highest AMR gene expression, while *agr* group III was most prevalent in animal samples and also exhibited elevated AMR gene expression within that group.

**Conclusions:** This study underscored the diverse distribution of AMR genes, with veterinarians and veterinary assistants facing higher occupational risks. The findings highlighted the importance of integrated antimicrobial stewardship and surveillance within a One Health framework to mitigate the spread of AMR in veterinary and community settings.

## 1. Introduction

Antimicrobial resistance (AMR) is a critical and growing global health threat, complicating the treatment of infectious diseases and leading to increased morbidity, mortality, and healthcare costs in human and veterinary medicine [1]. *Staphylococcus aureus*, a versatile pathogen, has developed resistance to multiple antibiotics, including methicillin-resistant *S. aureus* (MRSA), posing significant challenges for healthcare systems across species [2]. The frequent use and misuse of antibiotics in medical and veterinary practices drive this resistance, creating reservoirs of AMR bacteria that can be transmitted across species [3]. AMR and MRSA have serious implications for public health because treatment protocols become less effective. *Staphylococcus aureus* is of particular concern owing to its ability to cause various infections, high potential for antibiotic resistance, and versatility as a pathogen that can colonize multiple hosts, including humans and animals [4, 5]. The One Health framework, emphasizing the interconnectedness of human, animal, and environmental health, has critical importance for understanding and addressing the growing AMR crisis. This approach underscores the need for integrated studies across sectors to develop comprehensive strategies for managing resistance [6]. The role of companion animals in transmitting AMR bacteria to humans is becoming increasingly relevant, particularly considering the growing number of pet owners and the close physical interactions between pets and their owners [7, 8]. Pets can act as reservoirs of AMR pathogens, facilitating transmission through direct contact or environmental contamination [9].

The veterinary setting, where antibiotics are frequently used, represents a key interface for studying AMR dynamics and zoonotic risks [10]. Veterinary services are widespread and pet ownership is high in Thailand; thus, investigating the prevalence of AMR in *S. aureus* among human and animal populations is essential. Previous studies have highlighted the prevalence of *S. aureus* and MRSA isolated from pets in various regions of Thailand, emphasizing the importance of ongoing surveillance and the characterization of resistant strains [11, 12]. Existing studies have focused on isolated regions or specific populations, such as pets, without comprehensively addressing AMR prevalence and transmission patterns of *S. aureus* among the broader veterinary community, including veterinarians, veterinary assistants, pet owners, and pets. In addition, there is a lack of updated region-specific data on AMR in *S. aureus* across multiple provinces. Therefore, there is a critical need for comprehensive, multiregional studies to assess the AMR prevalence and characteristics of *S. aureus* in veterinary practices across Thailand, contributing to the broader understanding of AMR transmission in the One Health context.

This study aimed to investigate the prevalence of AMR in *S. aureus* isolated from humans (veterinarians, veterinary assistants, and pet owners) and animals (dogs and cats) visiting veterinary practices across five provinces in Thailand. By analyzing antimicrobial susceptibility and resistance genes, such as *bla*Z (β-lactam resistance), *mec*A (methicillin resistance), and *aac*A*–aph*D (aminoglycoside resistance), this study provided insights into the mechanisms driving resistance in these populations. The prevalence of accessory gene regulator (*agr*) groups, which influence virulence and resistance, was explored to better understand their distribution across host categories. These findings contribute to the development of effective infection control and antimicrobial stewardship strategies, protecting animal, and human health within the One Health framework.

## 2. Material and Methods

### 2.1. Sample Collection

The study design, including location selection, and sampling strategy, was based on methodologies described in previous reports [13]. In this study, samples were collected from humans (veterinarians, veterinary assistants, and pet owners) and animals (dogs and cats visiting veterinary premises). The selected veterinary clinics (without in-patient facilities) and hospitals (with in-patient care) were located in five provinces across different regions of Thailand, namely Nakhon Pathom (central), Nakhon Ratchasima (northeastern), Chon Buri (eastern), Chumphon (southern), and Chiang Mai (northern). These locations aligned with those from a previous study [13]. The sample size was calculated using Cochrane's formula [14] with a 95% confidence level, 5% margin of error, and desired prevalence of 0.5. Study sites were purposively selected with formal permission and voluntary participation. The number of samples was proportionally determined according to the population sizes and the ratios of dogs and cats in each province. Veterinary personnel from each facility, along with animal owners, were invited to participate in the study.

This study on pet owners was approved by the Mahidol University Central Institutional Review Board (COA No. MU-CIRB 2019/099.0706). Human participants provided consent by signing forms for sample collection. The animal study was reviewed and approved by The Committee on the Care and Use of Laboratory Animals in the Faculty of Veterinary Science, Mahidol University, Thailand (approval no. MUVS-2019-02-12). Written informed consent was obtained from the owners for the participation of their animals in this study.

To analyze *S. aureus*, swabs were taken from the skin and/or ears of animals with skin and/or ear lesions (indicating bacterial infections) and from the nasal cavities of humans (indicating bacterial colonization). The samples were preserved in transport medium (Oxoid, UK) at 4°C and delivered to the Veterinary Biochemistry Laboratory, Faculty of Veterinary Sciences, Mahidol University, Thailand within 72 h of collection. Subsequently, *S. aureus* was cultured for further identification. *Staphylococcus aureus* was cultured for further identification. Subsequently, antimicrobial susceptibility testing was performed. For *S. aureus* identification, the specimens were inoculated on sheep blood and mannitol salt agar (Oxoid, UK) and incubated at 37°C for 24–48 h. After incubation, the suspected bacterial colonies were selected and identified by conventional methods, such as Gram staining (Merck, Germany), catalase testing (Merck, Germany), mannitol (Merck, Germany) and trehalose (Merck, Germany) fermentation, and coagulase production (Ramel; Oxoid, UK).

### 2.2. Antimicrobial Susceptibility Testing

Antimicrobial susceptibility testing and interpretation were conducted using the minimum inhibitory concentration method per the Clinical and Laboratory Standards Institute guidelines. Specifically, M100 [15] was used for *S. aureus* isolates from humans, and VET01S [16] was applied for *S. aureus* isolates from animals.

All *S. aureus* isolates were examined for susceptibility to the following antimicrobial classes using a commercial Sensititreä Companion Animal Gram Positive COMPGP1F Vet AST Plate: β-lactams (ampicillin, penicillin, oxacillin + 2% NaCl, amoxicillin-clavulanic acid, cephalothin, cefazolin, and cefpodoxime), fluoroquinolones (enrofloxacin, marbofloxacin, and pradofloxacin), glycopeptides (vancomycin), aminoglycosides (amikacin and gentamicin), macrolides (erythromycin), tetracyclines (doxycycline and minocycline tetracycline), lincosamides (clindamycin), folate pathway antagonists (trimethoprim/sulfamethoxazole), nitrofurans (nitrofurantoin), phenicols (chloramphenicol), and ansamycins (rifampin). Antimicrobial susceptibility testing was performed according to the manufacturer's protocol. *Staphylococcus aureus* isolates from humans were further tested for susceptibility to norfloxacin and ciprofloxacin (both from Sigma–Aldrich, St. Louis, MO, USA). *S. aureus* ATTC-29,213 was used as the control strain.

### 2.3. Multiple Antibiotic Resistance (MAR) Index Calculation

The MAR index quantifies an isolate's resistance to multiple antibiotics, helping assess the risk and extent of antibiotic resistance in microbial populations. The MAR index was calculated as *a*/*b*, where *a* is the number of antibiotics to which an isolate is resistant, and *b* is the total number of antibiotics tested [17].

### 2.4. Detection of AMR and Agr Genes

Bacterial genomic DNA extraction was performed using the DNA extraction kit (Geneaid, Taiwan). Bacterial cells were lysed by adding the lysis buffer. In the extraction column, DNA adhered to the membrane and was subsequently eluted using an elution buffer. The quantity of extracted DNA was determined by measuring the absorbance at 260 nm using a spectrophotometer. AMR genes (*bla*Z, *mec*A, *aac*A*–aph*D, msrA, *tet*K, gyrA, *grl*A, *dfr*G, and *cfr*) [18] and *agr* [19] were identified using polymerase chain reaction (PCR). The total PCR mixture volume was 25 µL, containing 1 µM of each AMR gene primer and 2 µM *agr* primer, 2.5 µL of 10 U Taq PCR buffer, 0.2 mM dNTP, 2 mM MgCl_2_, and 1 U Taq DNA polymerase (Thermo Scientific, Germany). The PCR mixture underwent the following thermal cycling conditions using the Flexcycler^2^ (Analytik Jena, Germany): an initial 5 min at 95°C, followed by 30 cycles of amplification at 95°C for 30 s, annealing at the temperature specified for each gene for 30 s, and extension at 72°C for 60 s, with a final extension at 72°C for 10 min. The amplified products were analyzed using 1.5% agarose gel electrophoresis and stained with SYBR Safe (Invitrogen, USA). DNA bands were visualized under an ultraviolet transilluminator (UVP Bioimaging System, Invitrogen).

### 2.5. Network and Statistical Analysis

An undirected two-mode weighted network was built, in which the nodes in the first mode represented antibiotics, and the ones in the second mode were the subjects, including humans, cats, and dogs. An edge showed the resistance between a pair of antibiotics and a subject. The weight of each node was calculated from degree centrality, which is the number of edges attached to the node [20]. The “igraph” package in program R was used in the network analysis and visualization. The occurrence of resistance was described using the percentage of isolates and relevant resistance.

## 3. Results

### 3.1. Sample Characteristics and Occurrence of *S. aureus*

**Figure 1:**
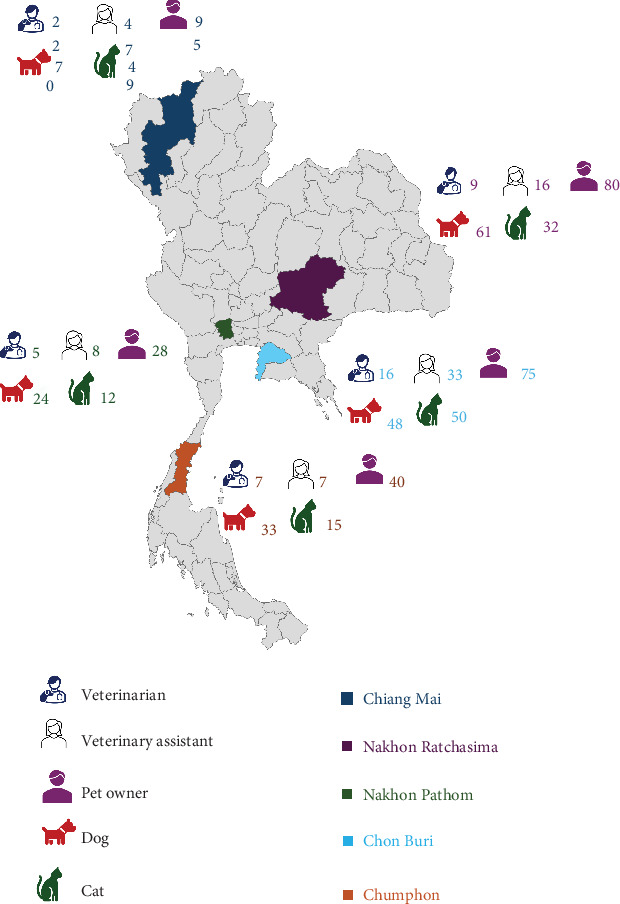
The map illustrates the distribution of samples isolated from humans and animals across the five sampling locations (provinces).

A total of 882 samples were collected across five provinces in Thailand. The northern region had the highest proportion of samples (32.09%), followed by the eastern (25.17%), northeastern (22.45%), southern (11.56%), and central (8.73%) regions (Figure 1). Skin or ear swabs were obtained from 236 dogs and 158 cats exhibiting cutaneous and otic lesions. Nasal swabs were collected from 318 owners, 59 veterinarians, and 111 veterinary assistants (Figure 1).

**Figure 2:**
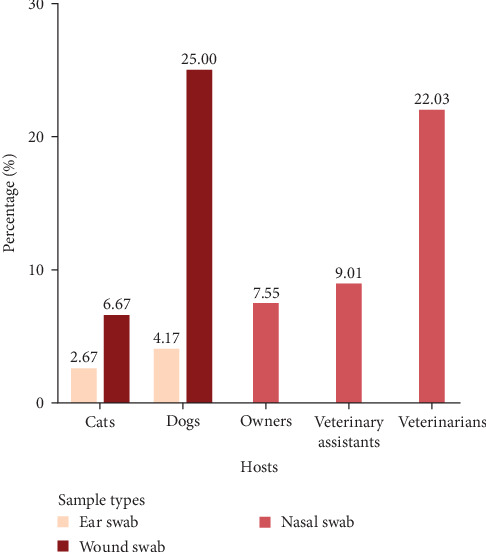
Percentage of *Staphylococcus aureus* in human and animal samples, categorized by sample type.

From these 882 samples, 188 *S. aureus* isolates were identified and used for further antimicrobial susceptibility and resistance gene analyses. These included isolates from veterinarians (*N* = 37), veterinary assistants (*N* = 27), pet owners (*N* = 65), dogs (*N* = 46), and cats (*N* = 13).  Table 1 summarizes the distribution of *S. aureus* isolates, calculated as the number of positive samples relative to the total number of collected samples, categorized by host. The highest prevalence of *S. aureus* in dogs and cats was observed in wound swabs, with rates of 25% and 6.67%, respectively (Figure 2).

### 3.2. AMR Profiles in *S. aureus* Across Veterinarians, Veterinary Assistants, Pet Owners, Dogs, and Cats

**Figure 3:**
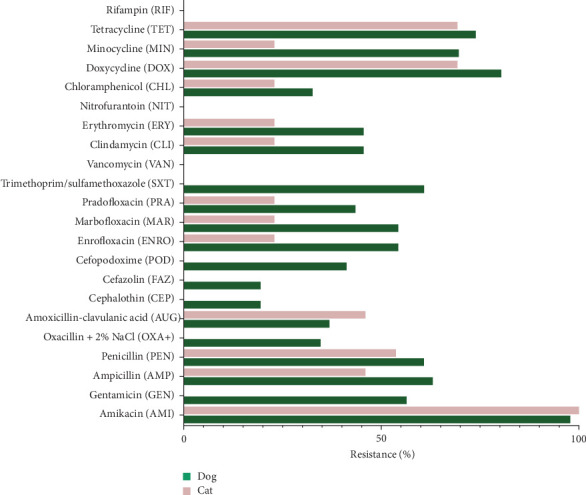
AMR profiles in *Staphylococcus aureus* isolates from pets.

Figure 3 shows the AMR profiles of *S. aureus* isolates from dogs and cats. Multiple antimicrobials were used in the study, and the proportion of resistance to each antimicrobial is shown, indicating significant variations between isolates from dogs and cats. For instance, isolates from dogs had significant resistance to most antimicrobials, whereas those from cats showed lower resistance. However, cat isolates had much greater amoxicillin-clavulanic acid resistance. Neither cat nor dog isolates showed resistance to rifampicin, nitrofurantoin, or vancomycin. MRSA isolates only found in dogs and not cats were resistant to oxacillin.

**Figure 4:**
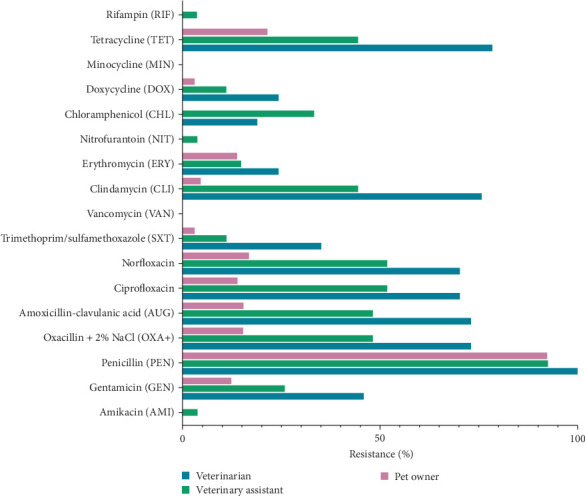
AMR profiles in *Staphylococcus aureus* isolates from humans.

Figure 4 indicates significant differences in AMR among veterinarians, veterinary assistants, and pet owners. Veterinarians had high resistance to tetracycline, chloramphenicol, trimethoprim/sulfamethoxazole, norfloxacin, ciprofloxacin, gentamicin, and amikacin. Although all groups had low resistance to rifampin and vancomycin, veterinarians still showed higher rates for many antibiotics than pet owners. Veterinary assistants had the highest resistance to minocycline, doxycycline, erythromycin, and clindamycin, followed closely by veterinarians. Minimal resistance to nitrofurantoin was observed in all groups but slightly higher in veterinarians. Overall, veterinarians had a higher AMR rate, emphasizing the need for targeted antimicrobial stewardship in this group.

### 3.3. High MAR in *S. aureus* Across Veterinarians, Veterinary Assistants, Pet Owners, Dogs, and Cats

**Figure 5:**
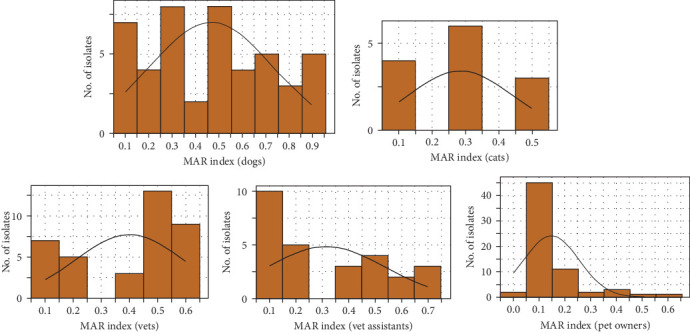
MAR index of *Staphylococcus aureus* isolated from dogs, cats, veterinarians, veterinary assistants, and pet owners.

The MAR index for *S. aureus* across veterinarians, veterinary assistants, pet owners, dogs, and cats were evaluated (Figure 5). The MAR index, calculated by the ratio of resistant antibiotics to the total antibiotics tested, ranged from 0.1 to 0.6 for veterinarians, with most isolates between 0.2 and 0.4. Veterinary assistants had a MAR index between 0.3 and 0.5, whereas pet owners had most isolates within 0.1–0.3. Dogs had a MAR index of 0.1–0.9, mostly between 0.3 and 0.5, whereas the MAR index of cats ranged from 0.1 to 0.5, concentrating between 0.2 and 0.4. *S. aureus* isolates showed high resistance, with 85% of dog isolates and 69% of cat isolates resistant to four or more drugs. Among humans, veterinarians had the highest resistance rate (70%) to four or more antimicrobials. The multidrug resistance (MDR) phenotype was prevalent in 82.61% of dog isolates, 69.23% of cat isolates, and 67.57% of veterinarian isolates, whereas 48.15% of the isolates were from veterinary assistants and 20% from pet owners. More than half (53.62%) of *S. aureus* isolates had a MAR index of >0.2, with the highest rates in dogs, followed by cats.

### 3.4. AMR Genes

The prevalence of AMR genes across various hosts (dogs, cats, veterinarians, veterinary assistants, and pet owners) was examined (Table 2). There were significant differences in the distribution of AMR genes related to resistance to β-lactams, aminoglycosides, quinolones, macrolides, lincosamides, tetracyclines, trimethoprim, and chloramphenicol. *bla*Z was highly prevalent in all groups, especially in veterinarians (100%) and veterinary assistants (92.59%). *mec*A was detected in veterinarians (72.97%) and dogs (34.78%), absent in cats, and low in pet owners (15.38%). *aac*A*–aph*D was found in 36.96% of dogs, 53.85% of cats, and lower in other groups. The quinolone resistance gene *gyr*A was high in veterinarians (64.86%) and dogs (54.35%), whereas *grl*A was lower across all groups, with dogs having the highest prevalence (17.39%). Macrolide resistance genes *msr*A and *erm*A were detected at low levels in all groups. The lincosamide resistance gene *lin*A was present in 34.78% of dogs and 23.08% of cats but absent in pet owners. The tetracycline resistance gene *tet*K was common in veterinarians (64.86%) and cats (53.85%). The accessory gene *agr*I was high in cats (69.23%) and veterinarians (72.97%), *agr*II was rare across most groups, *agr*III was predominant in dogs (93.48%), and *agr*IV was generally low but higher in veterinary assistants (37.04%). The study highlighted the diverse distribution of AMR genes, with veterinarians and veterinary assistants having a higher prevalence for several resistance genes than pet owners and animals.

### 3.5. Network Analysis

**Figure 6:**
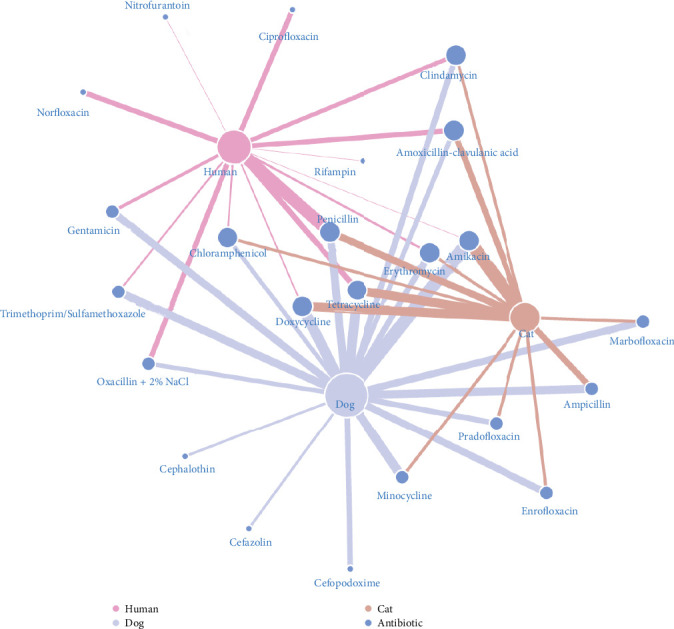
AMR network in *Staphylococcus aureus* among humans, dogs, and cats in veterinary care environments. The node size indicates the proportional degree of centrality, the width of the edges shows the relative resistance rate, and the color of the nodes and edges differentiates animal species from antibiotics.

In Figure 6, the number of antibiotics resisted by *S. aureus* in humans, dogs, and cats was 14, 19, and 13 (from the 24 tested antibiotics), respectively (represented by the degree centrality of the nodes). Resistance to eight antibiotics each was observed in three and two species, whereas the other seven were resisted by only one species. Nevertheless, vancomycin was not resistant to *S. aureus* in any species. About 94.57% of human isolates were resistant to penicillin, followed by tetracycline (42.64%) and norfloxacin (39.53%). In dogs, *S. aureus* was resistant to amikacin (97.83%), doxycycline (80.43%), and tetracycline (73.91%). Like in dogs, amikacin was most resistant (100%), followed by doxycycline and tetracycline (both 69.23%) in cats.

## 4. Discussion

AMR in *S. aureus*, especially MRSA, poses a substantial threat to human and animal health, as it limits treatment options and increases morbidity and mortality [2, 21]. In veterinary medicine, AMR complicates treatment, increases healthcare costs, and prolongs animal suffering. Pets act as reservoirs for resistant bacteria, facilitating transmission between animals and humans, which further elevates public health risks [5, 22]. Effective antibiotic stewardship is essential to mitigate these challenges [22, 23]. This study analyzed the prevalence of AMR in *S. aureus* isolates from humans (veterinarians, veterinary assistants, and pet owners) and animals (dogs and cats) across five provinces in Thailand, highlighting the need for targeted interventions to control the spread of resistant strains. The findings demonstrated the complex dynamics of AMR within veterinary settings and its implications for public health.

The highest prevalence of *S. aureus* was observed in veterinarians (22.03%), followed by veterinary assistants (9.01%), dogs (7.2%), pet owners (7.55%), and cats (3.16%). Similarly, previous studies reported that individuals in close contact with animals are more likely to harbor *S. aureus* [23, 24]. Antimicrobial susceptibility testing revealed substantial antibiotic resistance across isolates, particularly concerning amikacin resistance in dogs and cats, β-lactam resistance in humans, and MRSA prevalence in veterinarians. These findings aligned with global trends indicating an increasing burden of AMR in *S. aureus* [25, 26]. Among animals, resistance to aminoglycosides, particularly amikacin, was considerable owing to selective pressure from veterinary use [3]. Dog isolates exhibited higher resistance across multiple antibiotic classes, whereas cat isolates had substantial resistance to amoxicillin-clavulanic acid. Most dog and cat isolates exhibited MDR, with dog isolates demonstrating notably high MAR indices. These trends raised concerns about managing pet infections, as MDR *S. aureus* could serve as a reservoir for resistance genes that could be transferred to other pathogens or even humans. Among humans, veterinarians had the highest resistance rates, particularly to methicillin, with 72.97% of isolates classified as MRSA, followed by veterinary assistants (48.15%) and pet owners (15.38%). This high MRSA prevalence among veterinarians highlighted their occupational risks related to repeated exposure to antimicrobial agents and *S. aureus* in clinical settings [27, 28]. The high MDR rate in veterinarians, with 57.46% of isolates having an MAR index of ≥0.5, signaled substantial infection risks and healthcare costs associated with resistant infections. This occupational exposure increased infection risks and healthcare costs, whereas veterinary assistants and pet owners exhibited substantial antibiotic resistance attributable to contact with resistant strains from animals even at low risk [7]. Variations in antibiotic use, genetics, and environmental factors across hosts may contribute to differences in resistance mechanisms [26]. In addition, network analysis illustrated that *S. aureus* in dogs had the highest resistance spectrum (19 antibiotics), followed by humans (14) and cats (13), with resistance overlapping across species for eight antibiotics and being host-specific for seven antibiotics. The high rates of resistance to penicillin (94.57%) in humans and amikacin in dogs (97.83%) and (100%), along with strong tetracycline and doxycycline resistance in animals, highlights interspecies similarities and host-specific selective pressures, emphasizing the need for a One Health approach to antimicrobial stewardship.

The detection of resistance genes, such as *bla*Z, *gyr*A, and *tet*K, across all host groups reflects the widespread dissemination of resistance mechanisms. *bla*Z, commonly associated with β-lactam resistance, was detected in all host categories, consistent with its frequent use in human and veterinary medicine [28]. *mec*A, conferring methicillin resistance, has been detected in several host species, and it might pose a zoonotic risk to humans, particularly among veterinarians (72.97%), indicating a substantial MRSA reservoir [29, 30]. The detection of *tet*K and *gyr*A reflected tetracycline and quinolone resistance, respectively [31, 32]. The high *gyr*A prevalence in dogs and veterinarians highlighted the common usage of quinolones in these groups, with implications for treating infections that necessitate such broad-spectrum antibiotics. *agr* typing in *S. aureus* revealed a diverse distribution of *agr* groups, each associated with different virulence and resistance patterns [33]. The *agr* system, a global regulator of staphylococcal virulence factors, is categorized into four groups (*agr*I–*agr*IV) [34]. In this study, *agr*I was the most prevalent group, particularly in humans; this is consistent with findings in certain regions. However, *agr*IV was rarely detected, suggesting geographical variation in the *agr* group distribution [34, 35]. These *agr* genes, particularly in veterinarians and pet owners, signal increased virulence and resistance, exacerbating the AMR crisis through potential cross-species transmission and environmental contamination.

The correlation between AMR phenotypes and corresponding AMR genes in *S. aureus* isolates highlights key resistance patterns across different host groups. The high prevalence of *bla*Z (100% in veterinarians and 92.59% in veterinary assistants) aligned with the widespread resistance to β-lactam antibiotics observed in these groups. Similarly, *mec*A was detected in veterinarians (72.97%) and dogs (34.78%), which is consistent with the presence of MRSA isolates exclusively in dogs. For aminoglycoside resistance, the presence of *aac*A–*aph*D in 36.96% of dogs and 53.85% of cats was linked to the observed resistance to gentamicin and amikacin, particularly in veterinarians. The high prevalence of *gyr*A in veterinarians (64.86%) and dogs (54.35%) supported the observed resistance to fluoroquinolones, such as ciprofloxacin and norfloxacin. Moreover, *tet*K was frequently detected in veterinarians (64.86%) and cats (53.85%), matching the high tetracycline resistance observed in these hosts. Notably, the MDR phenotype, prevalent in 82.61%, 69.23%, and 67.57% of dog, cat, and veterinarian isolates, respectively, aligned with the detection of multiple resistance genes in these groups. The presence of *agr*III in 93.48% of dog isolates suggested potential associations with virulence regulation and adaptation in this host. Overall, these findings indicate a strong correlation between phenotypic resistance and the presence of corresponding resistance genes, emphasizing the need for continuous surveillance and antimicrobial stewardship in veterinary and public health settings.

Although this study offers important insights, it had multiple limitations. The cross-sectional design restricts the ability to establish causal relationships between animal contact and AMR acquisition. The geographic scope of this study was limited to five provinces in Thailand, which may limit the generalizability of the findings to other regions. Future research should focus on the dynamics of *S. aureus* transmission between humans and animals in Thailand, with longitudinal studies providing deep insights into AMR trends over time. Investigating specific mechanisms of antibiotic resistance and the role of *agr* groups in virulence is also critical for developing targeted interventions. Pet animals can carry and transmit AMR bacteria to humans through direct contact or environmental exposure, raising public health concerns, especially in veterinary settings. However, this study did not determine the direction of transmission. Further research is needed to understand how AMR spreads between humans and animals. This study revealed critical concerns for occupational health, antibiotic efficacy in pets, and the zoonotic potential of AMR in *S. aureus*, particularly MRSA. The high AMR prevalence among veterinarians and veterinary assistants, with 72.97% and 48.15% harboring MRSA, underscored their health risks, which can lead to difficult-to-treat infections, prolonged illness, and increased healthcare costs. AMR threatens the effectiveness of treatments in pets, as resistance in *S. aureus* isolates, especially MDR in dogs (82.61%) and cats (69.23%), complicates infection management. Furthermore, pets act as reservoirs for resistant bacteria, facilitating the spread of AMR between animals and humans. The detection of common resistance genes, such as *bla*Z, *mec*A, and *tet*K, and the high prevalence of *agr* genes in veterinarians and pet owners underscore the need for effective antibiotic stewardship, targeted interventions, and ongoing surveillance to combat the AMR crisis across human and animal populations within a One Health framework.

## 5. Conclusions

This study highlights the prevalence of *S. aureus* and AMR across various human and animal populations in Thailand, underscoring the interconnected nature of AMR transmission between species. The patterns observed suggest potential zoonotic transmission routes, particularly in veterinary settings, where close human–animal interactions facilitate the spread of resistant strains. Continuous surveillance of AMR trends and deep investigation into the genetic mechanisms driving resistance are essential to inform effective control strategies. Diverse *agr* groups were identified in *S. aureus*, each linked to specific virulence and AMR profiles. *Agr* group I was predominant in human isolates, showing the highest AMR gene expression, while *agr* group III was most prevalent in animal isolates and exhibited the highest AMR expression within that group. To mitigate this growing public health threat, implementing responsible and judicious antibiotic practices in human and veterinary medicine is crucial. These efforts must be guided by the One Health approach, recognizing the importance of integrated measures that protect the health of animals, humans, and the environment.

## Figures and Tables

**Table 1:** Distribution of *S. aureus* by host category and region.

Host category	No. of *S. aureus* isolates	Positive samples (%) (95% CI)	Contributing province (No. of *S. aureus* isolates)
Veterinarians (nasal swab)	37	22.03 (13.35–34.13)	Nakhon Pathom (3) Nakhon Ratchasima (3) Chon Buri (7) Chumphon (3) Chiang Mai (21)

Veterinary assistants (nasal swab)	27	9.01 (4.96–15.79)	Nakhon Pathom (6) Nakhon Ratchasima (13) Chon Buri (3) Chumphon (0) Chiang Mai (5)

Pet owners (nasal swab)	65	7.55 (5.12–10.98)	Nakhon Pathom (9) Nakhon Ratchasima (31) Chon Buri (17) Chumphon (3) Chiang Mai (5)

Dogs (ear or skin swab)	46	7.2 (4.54–11.23)	Nakhon Pathom (2) Nakhon Ratchasima (23) Chon Buri (6) Chumphon (3) Chiang Mai (12)

Cats (ear or skin swab)	13	3.16 (1.35–7.19)	Nakhon Pathom (0) Nakhon Ratchasima (9) Chon Buri (1) Chumphon (0) Chiang Mai (3)

**Table 2:** AMR genes of *S. aureus* isolated from dogs, cats, veterinarians, veterinary assistants, and pet owners.

AMR genes	Dogs (*n* = 46)	Cats (*n* = 13)	Veterinarians (*n* = 37)	Veterinary assistants (*n* = 27)	Pet owners (*n* = 65)
	*n* (%, 95% CI)	*n* (%, 95% CI)	*n* (%, 95% CI)	*n* (%, 95% CI)	*n* (%, 95% CI)
β-Lactam					
* bla*Z	28 (60.87, 46.45–73.60)	7 (53.85, 29.14–76.79)	37 (100, 90.59–100.00)	25 (92.59, 76.63–97.94)	60 (92.31, 83.22–96.66)
* mec*A	16 (34.78, 22.68– 49.22)	0 (0.00, 0.00–22.80)	27 (72.97, 57.02–84.60)	13 (48.15, 30.74–66.01)	10 (15.38, 8.57–26.05)
Aminoglycoside					
* aac*-*aph*D	17 (36.96, 24.52–51.39)	7 (53.85, 29.14–76.79)	12 (32.43, 19.63–48.53)	3 (11.11, 3.85–28.05)	4 (6.15, 2.41–14.78)
Quinolone					
* gyr*A	25 (54.35, 40.18–67.84)	3 (23.08, 8.17–50.25)	24 (64.86, 48.75–78.17)	14 (51.85, 33.98–69.25)	11 (16.92, 9.72–27.81)
* grl*A	8 (17.39, 9.08–30.72)	1 (7.69, 1.37–33.31)	1 (2.7, 0.47–13.82)	3 (11.11, 3.85–28.05)	9 (13.85, 7.45–24.26)
Macrolide					
* msr*A	2 (4.35, 1.20– 14.53)	1 (7.69, 1.37–33.31)	0 (0.00, 0.00–9.40)	1 (3.7, 0.65–18.28)	3 (4.62, 1.58–12.71)
* erm*A	1 (2.17, 0.38–11.33)	1 (7.69, 1.37–33.31)	6 (16.22, 7.65–31.13)	3 (11.11, 3.85–28.05)	3 (4.62, 1.58–12.71)
Lincosamide					
* lin*A	16 (34.78, 22.68– 49.22)	3 (23.08, 8.17–50.25)	6 (16.22, 7.65–31.13)	3 (11.11, 3.85–28.05)	0 (0.00, 0.00–5.58)
Tetracycline					
* tet*K	20 (43.48, 30.20–57.75)	7 (53.85, 29.14–76.79)	24 (64.86, 48.75–78.17)	8 (29.63, 15.85–48.48)	14 (21.54, 13.28–32.96)
Trimethoprim					
* dfr*G	19 (41.3, 28.28–55.66)	0 (0.00, 0.00–22.80)	1 (2.7, 0.47–13.82)	3 (11.11, 3.85–28.05)	0 (0.00, 0.00–5.58)
Chloramphenicol					
* cfr*	1 (2.17, 0.38–11.33)	1 (7.69, 1.37–33.31)	1 (2.7, 0.47–13.82)	3 (11.11, 3.85–28.05)	0 (0.00, 0.00–5.58)
Accessory gene					
* agr*I	0 (0.00, 0.00–7.70)	9 (69.23, 42.36–87.31)	27 (72.97, 57.02–84.60)	3 (11.11, 3.85–28.05)	27 (41.54, 30.36–53.66)
* agr*II	0 (0.00, 0.00–7.70)	0 (0.00, 0.00–22.80)	0 (0.00, 0.00–9.40)	1 (3.7, 0.65–18.28)	8 (12.31, 6.37–22.45)
* agr*III	43 (93.48, 82.49–97.75)	0 (0.00, 0.00–22.80)	4 (10.81, 4.28–24.70)	9 (33.33, 18.64–52.17)	14 (21.54, 13.28–32.96)
* agr*IV	0 (0.00, 0.00–7.70)	4 (30.77, 12.68–57.63)	0 (0.00, 0.00–9.40)	10 (37.04, 21.53–55.77)	2 (3.08, 0.84–10.54)
ND	3 (6.52, 2.24–17.50)	0 (0.00, 0.00–22.80)	6 (16.22, 7.65–31.13)	4 (14.82, 5.91–32.47)	14 (21.54, 13.28–32.96)

## Data Availability

The data that support the findings of this study are available from the authors upon reasonable request.
